# Supplementary material for: Slab steepening and rapid mantle wedge replacement during back-arc rifting in the New Hebrides
Source: Nat Commun. 2024 Jul 18;15:6070. doi: 10.1038/s41467-024-50445-3 (PMC11258315; doi:10.1038/s41467-024-50445-3)

Supplementary Figure 1. Results of the Ar/ar age dating for the Futuna Trough lavas. Panels show the K/Ca ratios
age in millions of years (Ma) for either groundmass or separates of plagioclase.

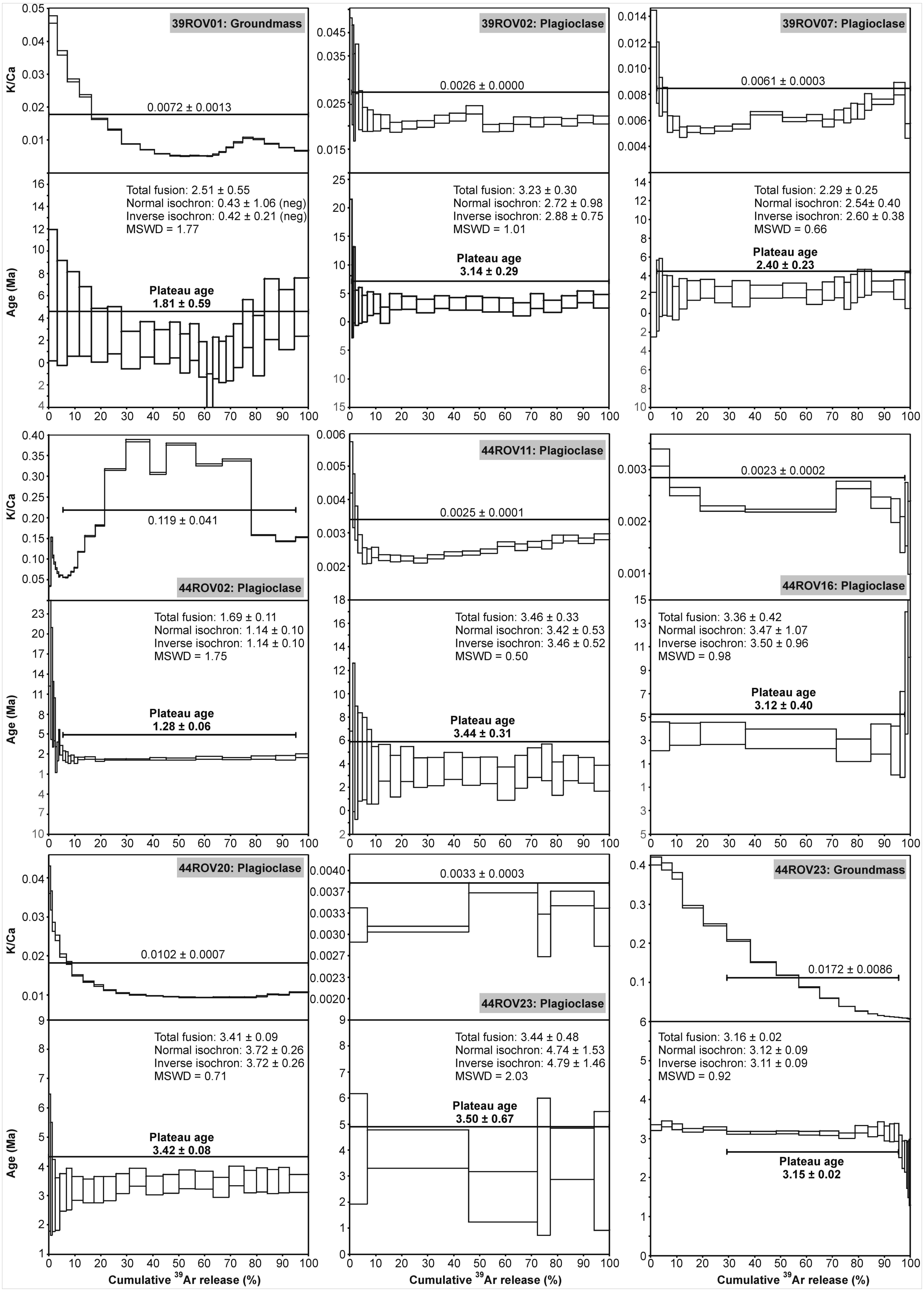

Supplement: Supplementary file 1 — Supplementary Information [file 41467_2024_50445_MOESM1_ESM.pdf]
