# Supplementary material for: Slab steepening and rapid mantle wedge replacement during back-arc rifting in the New Hebrides
Source: Nat Commun. 2024 Jul 18;15:6070. doi: 10.1038/s41467-024-50445-3 (PMC11258315; doi:10.1038/s41467-024-50445-3)
Supplement: Supplementary file 3 — Description of Additional Supplementary Files [file 41467_2024_50445_MOESM3_ESM.pdf]

Description of Additional Supplementary Files

Supplementary Data 1: Major and trace element compositions of the Futuna Trough lavas determined by XRF (whole rock powders), electron microprobe (volcanic glass), and ICP-MS. Results for international rock standards analyzed with the samples are also shown.

Supplementary Data 2: Age data of selected Futuna Trough lavas determined by the 40Ar/39Ar method at Oregon State University (OSU), USA.
